# Comparison of mortality between peritoneal dialysis and hemodialysis in polycystic kidney disease

**DOI:** 10.1080/0886022X.2018.1487867

**Published:** 2019-01-16

**Authors:** Xingxing Fang, Meizi Kang, Dongmei Chen, Lianglan Shen

**Affiliations:** Department of Nephrology, First People's Hospital of Nantong, Nantong, ChinaShen.llan@163.com

Dear Sir,

Polycystic kidney disease (PKD) is characterized by progressive growth of cysts. PKD affects about 1‰ people worldwide [1,2]. PKD is the fourth leading cause of renal replacement therapy (RRT) [2]. RRT for PKD includes hemodialysis (HD) and peritoneal dialysis (PD) [3]. PD was used less frequently than HD in PKD patients [4]. PD was avoided for PKD patients due to enlarged kidneys, intraperitoneal pressure, and complications. Evidences about the mortality risk between PD and HD in PKD are controversial and unresolved. Therefore, we used meta-analysis to illustrate whether the mortality is different among individuals receiving PD comparing HD.

**Figure 1.**
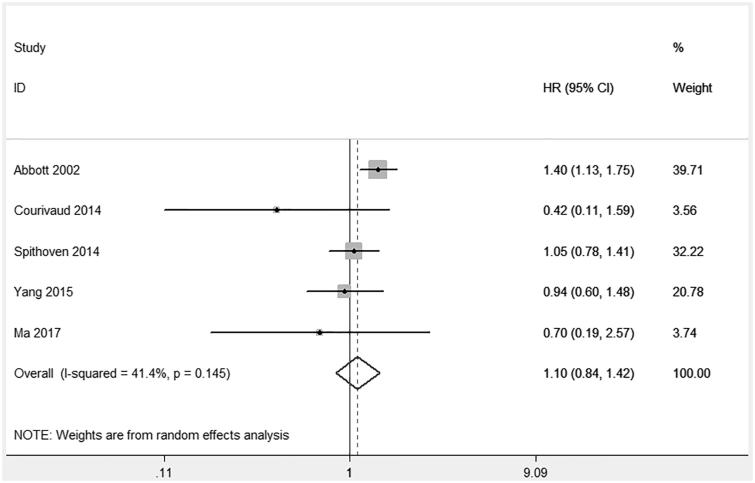
Mortality comparing HD with PD in PKD patients.

We searched PubMed, EMBASE, Web of Science, and Cochrane Database (until April 2018) about clinical studies involving PKD patients comparing PD and HD. Hazard ratios (HRs) were pooled with 95% confidence intervals (CIs) for dichotomous data. Primary outcome was the mortality. Statistical heterogeneity was evaluated using *I*^2^ statistics. Risk of bias ratings for each study were assessed with the Newcastle–Ottawa Quality Assessment Scale.

The process of selecting relevant studies found 126 publications from databases. Finally, five cohort studies involving 7593 PKD patients were identified [4–8]. The risk of bias evaluation of each study was more than six stars representing low risk of bias. There were no significant differences in mortality (HR: 1.10, 95% CI: 0.84–1.42, *p* = .49; *I*^2^ = 41%, *p* = .15, Figure 1) between HD and PD. Publication bias was negative (Begg’s test *p* = .22; Egger’s test, *p* = .07).

The result indicates that there is no significant difference of mortality between PD and HD in PKD patients. PD may be as safe as HD in survival for PKD patients receiving RRT. Further studies evaluating dialysis technical survival should be undertaken.
